# Nurses’ knowledge, attitudes and practices regarding evidence-based practice in the prevention of mother-to-child transmission of HIV programme in Malawi

**DOI:** 10.4102/curationis.v40i1.1656

**Published:** 2017-04-12

**Authors:** Chisomo Mulenga, Joanne R. Naidoo

**Affiliations:** 1 School of Nursing and Public Health, Howard College Campus, University of KwaZulu-Natal, South Africa

## Abstract

**Background:**

HIV continues to be a global public health concern with Malawi being among the worst affected countries. The prevalence of HIV among pregnant women is also very high, thereby raising concerns of mother-to-child transmission of the virus. Prevention of mother-to-child transmission (PMTCT) of HIV is therefore a priority in the efforts to curb the HIV pandemic. Keeping in mind that the area of HIV management is rapidly evolving, underpinning nursing care with evidence-based practice is essential and has been reported to reduce mother-to-child transmission.

**Objectives:**

The aim of the study was to explore and describe the knowledge, attitudes and practices of nurses regarding evidence-based practice in PMTCT at a selected hospital in Malawi.

**Methods:**

An exploratory descriptive quantitative design was used, and 81 nurses working in paediatric, obstetrics and gynaecology departments completed a self-administered questionnaire. Data were analysed using Predictive Analytics Software.

**Results:**

The results showed that nurses had average knowledge of evidence-based practice and although their attitudes were favourable, their practice was very low. Certain sociodemographic variables had an influence on the respondent’s knowledge, attitudes and practices. Furthermore, the results have indicated that evidence-based practice was mainly hampered by insufficient resources and difficulties in accessing research articles. It emerged from the study that mentoring, training and access to literature could facilitate evidence-based practice in PMTCT among nurses.

**Conclusion:**

Nurses need to be provided with the necessary support including education and resources if evidence-based practice in PMTCT is to be promoted.

## Introduction

HIV continues to be a global public health concern with an estimated 36.9 million people living with the virus in 2014 (Joint United Nations Programme on HIV/AIDS [UNAIDS] 2015). Sub-Saharan Africa (SSA), home to a third of the world’s population, bears 70% of all HIV infections having 25.8 million infected people (UNAIDS 2015). Women worldwide remain the most vulnerable group, with an estimated HIV prevalence of 52% (UNAIDS 2013a). According to UNAIDS (2013b), in 2012, SSA harboured 92% of all HIV-infected women and 90% of all HIV-infected pregnant women. HIV prevalence among children is also high in SSA. It is estimated that in 2012, 90% of all children who had acquired the virus were from SSA (UNAIDS 2013a).

Malawi, like other SSA countries, is badly affected by the HIV pandemic with a national prevalence of 10.6% among adults aged 15–49. Women have the highest burden, with an estimated HIV prevalence of 12.9% compared to 8.1% for men. HIV prevalence among pregnant women is estimated to be 8.8%, which is also high (Malawi Demographic Health Survey 2010). Mother-to-child transmission (MTCT) of HIV is the second major mode of transmission and accounts for approximately 25% of all new infections (Ministry of Health 2012). It is estimated that, without interventions, transmission rates range from 15% to 45%. However, the MTCT rate can be reduced to levels below 5% with the implementation of effective prevention of mother-to-child transmission (PMTCT) of HIV interventions (World Health Organization [WHO] 2013). PMTCT is therefore considered a priority in the global efforts to curb the HIV pandemic (Govender & Coovadia 2014).

Nurses are the major providers of PMTCT services in SSA. The nurses perform a wide variety of PMTCT-related tasks that range from HIV diagnosis, prescription of anti-retroviral therapy (ART), ensuring safe obstetrics during delivery, counselling on infant feeding and management of opportunistic infections (Zachariah et al. 2009). PMTCT knowledge and guidelines, however, are rapidly changing as more research evidence emerges. For example, the WHO infant feeding guidelines have evolved over the last 25 years and at least 16 documents have been produced to serve as guidelines for HIV-infected women (Moland et al. 2010). Thus, nurses need to possess the most up-to-date knowledge through continuous training. This, however, is a challenge in Malawi (Chimwaza et al. 2014).

Previous studies on knowledge and practices of PMTCT among nurses have highlighted knowledge deficit in various aspects of the PMTCT cascade. A rapid assessment of infant feeding policies and programmes in four African countries (Botswana, Kenya, Malawi and Uganda) shows a high PMTCT established knowledge deficit among nurses on risks of HIV transmission with respect to breast feeding and infant feeding options (Chopra & Rollins 2009). Similarly, a cross-sectional survey of 231 Nigerian nurses on the translation of research into practice in PMTCT established that nurses’ practices related to intrapartum care of the mother, infant feeding and care of the neonate were not in accordance with evidence-based guidelines recommendations (Ogbolu et al. 2013). Du-Preez, Du-Plessis and Pienaar (2006) surveyed 31 South African midwives on their knowledge of safe intra-partum practices and established certain inconsistencies. The midwives had adequate knowledge in areas such as CD4 count, HIV testing during labour, and recommended mode of delivery for HIV-infected women. However, they demonstrated uncertainties with respect to most of the safe intra-partum practices that limit MTCT of HIV (Du-Preez et al. 2006). Lack of training in PMTCT was the main reason cited in all these studies for the poor knowledge and gaps in practice (Chopra & Rollins 2009; Du-Preez et al. 2006; Ogbolu et al. 2013). Consequently, the nurses base their PMTCT practices on advice of colleagues, experience, rituals and traditions, which on their own cannot improve the quality of care (Nguyen et al. 2009; Salyer, Walusimbi & Fitzpatrick 2008).

PMTCT being a field that is rapidly evolving, the nurses need to be abreast of current developments. As continuous training is a challenge for these nurses, they are expected to embrace evidence-based practice (EBP) as there is compelling evidence that successful implementation of evidence-based interventions from empirical research can result in remarkable reduction of MTCT of HIV. In the United Kingdom and Ireland, an overall MTCT rate of 1.2% was recorded with the successful implementation of evidence-based PMTCT interventions (Townsend et al. 2008). In SSA, evidence-based PMTCT interventions also prevented 350 000 new HIV infections in children (Govender & Coovadia 2014). Scott and McSherry (2009), however, contend that for EBP to occur, nurses need to be aware of what EBP is, what it constitutes and the processes to engage with and apply the evidence. There is, however, a dearth of literature on EBP among nurses working in PMTCT programmes. It was therefore critical to assess if the PMTCT nurses were aware of and engaging in EBP.

### Problem statement

HIV is a field that is rapidly evolving as more research evidence continues to emerge (Wall 2014). Despite numerous studies being undertaken, the HIV pandemic remains high in Malawi with women being the most severely affected group. This increases the risk of MTCT should the infected woman fall pregnant (Ministry of Health 2012). Nurses are the backbone of health service provision in Malawi. In many European countries, nursing education shifted from basic to degree programmes to prepare nurses for the requirements of EBP (Rudman et al. 2012). In Malawi, however, the major providers of PMTCT services are nurses, midwives or technicians who are trained below first degree level. The nurses rely on pre-service education, which is not standardised (Zuber et al. 2014), and in-service education, which is not available to all the nurses and is not done on a regular basis (Chopra et al. 2009). No studies have been conducted to explore EBP among these PMTCT nurses in Malawi.

## Objectives of the study

The study objectives were to explore nurses’ level of knowledge regarding EBP in PMTCT, nurses’ attitudes towards EBP in PMTCT, nurses’ practices in relation to EBP in PMTCT, the inter-relationships between nurses’ sociodemographic variables and their knowledge, attitudes and practices, and nurses’ perceptions of barriers and facilitators affecting EBP in PMTCT.

## Definition of key concepts

Evidence-based practice (EBP) is a process in which knowledge, or specifically clinical research findings or best available evidence, is supplemented by clinical expertise and patient preferences and incorporated into practice settings (Smith & Donze 2010).

Nursing in Malawi: A nurse is a person who has completed a programme of basic education and is qualified and authorised in their country to practise nursing (International Council of Nurses 2002). For the purpose of this study, the term ‘nurse’ was limited to those involved in rendering PMTCT services in Malawi (maternity, gynaecology and paediatric nurses) and was used interchangeably with ‘midwife’ as the majority of nurses in Malawi are also midwives. The nurses involved in PMTCT services in Malawi were of different categories, as described below:

- Professional nurses are nurses who successfully completed a four-year degree course in nursing (Nurses and Midwives Council of Malawi 2011).
- Registered nurse midwives are nurses who successfully completed a two-year upgrading diploma course in nursing and midwifery (Nurses and Midwives Council of Malawi 2011).
- Nursing midwifery technicians (NMTs) are nurses who successfully completed a three-year college-integrated programme in both nursing and midwifery in Malawi (Nurses and Midwives Council of Malawi 2011)

PMTCT is a comprehensive approach to combating HIV in infants and young children, specifically by reducing the risk of MTCT. It addresses a wide range of prevention, care, treatment and support services along the continuum of care from pregnancy through childhood (Global Fund 2010).

## Significance of the study

In its plan to eliminate MTCT of HIV, the Malawi government prioritised the development of healthcare providers’ capacities (Ministry of Health 2012). The present study, therefore, highlights the nurses’ current knowledge, attitudes and practices regarding EBP in PMTCT as EBP is one of the competencies required for nurses working with HIV-infected people (Relf et al. 2011).

## Research method and design

A non-experimental exploratory descriptive design was used for the study. The design was selected because it allows one to fully investigate the nature of the phenomenon and factors related to it. Additionally, there is no manipulation of the independent variables, and hence it gives a true reflection of the phenomena under study (Burns & Grove 2009).

### Population and sampling

The study was conducted at one of the tertiary hospitals in Malawi. The hospital has a bed capacity of 930 and serves approximately 5 million people. It has a number of departments; however, the study focused on the obstetrics, gynaecology and paediatrics departments where PMTCT services are provided. Purposive sampling was used to enrol only nurses working in these departments. To be eligible for inclusion in the study, nurses had to be working in the above departments and be permanently employed. Based on the inclusion criteria, a total of 86 nurses made up the target population; all nurses were approached to be part of the study and a total of 81 respondents constituted the final sample.

### Research instrument

The questionnaire was based on the following literature reviews: Upton and Upton (2006) on items for knowledge or skills and attitudes, Melnyk, Fineout-Overholt and Mays (2008) on practice, Gerrish et al. (2008) on barriers and Majid et al. (2011) on facilitators to EBP. The researcher added a section on sociodemographic data. Fourteen statements assessed knowledge or skills on a five-point Likert scale, with responses ranging from ‘poor’ as score 1 to ‘excellent’ as score 5. Scoring consisted of summing the responses for a minimum score of 14 and maximum score of 70.

The attitude scale consisted of four items on a five-point Likert scale ranging from ‘strongly agree’ as score 1 to ‘strongly disagree as score 5. Scoring for the items involved adding up the responses to a minimum score of four and maximum score of 20. Seventeen questions assessed the nurses’ practices on a five-point frequency scale ranging from zero times as score 0, to more than eight times as score 4. Scoring of the items involved summing the responses to the 17 items for a total score ranging from 0 to 68. The barriers scale had 14 items, and 6 items assessed the facilitators on a five-point Likert scale ranging from strongly agree to strongly disagree. The alternatives were collapsed into three categories of agree, disagree and neutral.

### Data collection procedure

Data for the study were collected during November 2014. An information sheet describing the details of the study was handed to the participants for informed consent. The information was also explained verbally. Those who gave consent were then handed questionnaires and were given guidance on how to complete the questionnaires. Participants were allowed to complete the questionnaires at their own convenience.

### Data analysis

Data were entered and subsequently analysed using the Predictive Analytics Software (PASW), version 21. Descriptive statistics such as frequencies, percentages, means and medians were used to summarise the data.

## Ethical considerations

Ethical clearance was obtained from the Biomedical Research Ethics Committee, University of KwaZulu-Natal, reference number BE386/14, and the National Health Sciences Research Committee, Malawi, reference number NHSRC #1314. Permission was also granted by the director of the selected hospital. The study had no physical, social or psychological risks. The participants were provided with all the information about the study verbally and in writing. Participation in the study was voluntary and no coercion was used. No identifying information was used on the questionnaires. The data were securely stored and were accessible to the researchers only.

## Results

The results are presented in line with the study objectives.

### Sociodemographic data

Most of the respondents (90%; *n* = 73) were female, and only 10% (*n* = 8) were male. The respondents’ ages ranged from 22 to 61 years with a median age of 35 years and interquartile range of 20 years. The majority of the respondents (61.7%; *n* = 50) were nursing midwifery technicians (NMTs). Professional nurses (those who successfully completed a four-year degree course in nursing) comprised only 29.6% (*n* = 24) and only 8.6% (*n* = 7) were registered nurses (those who successfully completed a two-year upgrading diploma course in nursing and midwifery). Analysis of qualification showed that the majority of the nurses were trained at a level below first degree as only 29% (*n* = 24) had qualified through a four-year bachelor degree programme. The respondents, work experience ranged from 1 to 36 years with a median experience of 10 years and an interquartile range of 15.5 years.

The majority of respondents (48.1%; *n* = 39) were working in the maternity department. Paediatrics comprised 39.5% (*n* = 32) the respondents and only 12.3% (*n* = 10) were working in the gynaecology department. The minimum length of time in a department was less than 1 year and the maximum was 34 years, with a median of 2.8 years and an interquartile range of 3 years. In terms of PMTCT training, the majority (70.4%: *n* = 57) of the participating nurses or midwives were trained. Of those who were trained, 73.7% (*n* = 42) had received in-service training and 31.6% (*n* = 18) had received pre-service education. Approximately half of the respondents (49.4%; *n* = 40) were trained in EBP. Of these, 52.5% (*n* = 21) received in-service training. Only 32.5% (*n* = 13) had received pre-service training and (15.0%; *n* = 6) had received both pre-service and in-service training.

### Knowledge, attitudes and practices of evidence-based practice in prevention of mother-to-child transmission

The results showed that the nurses possessed average knowledge about EBP in PMTCT with a mean (M) knowledge score of 39.2 out of a possible 70 with a standard deviation (SD) of 8.1. The individual item scores presented in Table 1 demonstrate that the respondents scored higher on: sharing of ideas with colleagues (*M* = 3.56; SD = 0.88), reviewing own practice (*M* = 3.54; SD = 0.86), dissemination of new ideas (*M* = 3.35; SD = 0.86), applying information to individual cases (*M* = 3.33; SD = 0.77), and identifying gaps in practice (*M* = 3.13; SD = 0.84). Most respondents had poor knowledge or skills in converting information needs into research questions (*M* = 2.20; SD = 0.95), research skills (*M* = 2.26; SD = 0.93), awareness of major information sources (*M* = 2.42; SD = 0.84), information technology (*M* = 2.46; SD = 0.93), and knowledge of how to retrieve evidence (*M* = 249; SD = 0.93).

**TABLE 1:** Knowledge, attitude and practice of evidence-based practice in prevention of mother-to-child transmission.

Item	*n*	Mean	SD
**Knowledge**			
Converting information needs to research questions	81	2.20	0.95
Research skills	81	2.26	0.93
Awareness of major information types/sources	81	2.42	0.84
Information technology skills	81	2.46	0.93
Knowledge of how to retrieve evidence	79	2.49	0.93
Ability to critically analyse evidence against set standards	81	2.53	0.98
Monitoring and reviewing of practice skills	81	2.64	0.89
Ability to determine how valid material is	81	2.65	0.85
Ability to determine how useful material is	81	2.91	0.86
Ability to identify gaps in professional practice	81	3.17	0.84
Ability to apply information to individual cases	80	3.33	0.77
Dissemination of new ideas about care to colleagues	81	3.35	0.86
Ability to review own practice	81	3.54	0.86
Sharing of ideas and information with colleagues	81	3.56	0.88
**Attitude**			
Workload too great to keep up to date with evidence	81	2.59	1.49
Recent clinical practice being questioned	81	3.26	1.11
Stick to tried and trusted methods rather than change	81	3.40	1.33
EBP is a waste of time	81	4.27	1.00
**Practice**			
Accessed the Cochrane database of systematic review	81	0.42	0.84
Critically appraised evidence from a research	81	0.42	0.70
Shared evidence from research with multidisciplinary team	81	0.47	0.77
Read and critically appraised clinical research	81	0.49	0.79
Informally discussed evidence from research	81	0.51	0.72
Used EBP guideline to change practice	81	0.57	0.75
Generated a Population Intervention Comparison Outcome (PICO) question about clinical practice	81	0.59	0.93
Shared evidence from a study in form of report/presentation	81	0.60	0.94
Shared evidence from a research study with patient/family	81	0.64	0.95
Evaluated the outcome of a practice change	81	0.81	0.92
Changed practice based on patient outcome data	81	0.86	0.97
Evaluated a care initiative by collecting patient outcome data	81	0.93	0.97
Shared EBP guidelines	81	0.99	1.13
Promoted the use of EBP to colleagues	80	1.00	1.10
Shared patient outcome data with colleagues	81	1.01	1.05
Used evidence to change clinical practice	81	1.02	1.07
Collected data on a patient problem	81	1.43	1.25

EBP, evidence-based practice.

The respondents demonstrated more favourable attitudes towards EBP with a mean score of 13.5 out of a possible 20, and a SD of 3.4. Workload being too great to keep up to date with new evidence received the lowest mean score of 2.59 (SD = 0.95). Even though the respondents had average knowledge and favourable attitudes towards EBP, they were implementing EBP to a limited extent. On a scale of 0–68, the median score was 11 and the interquartile range was 15. The individual item scores displayed in Table 1 show that on a scale of 1–5, the nurses’ highest practice mean score was one that was very low. Just as the majority of the nurses demonstrated poor knowledge or skill in research, they also had very low practice scores on research-related activities such as: accessing the Cochrane database of systematic review (*M* = 0.42; SD = 0.84), critically appraising PMTCT evidence from research (*M* = 40.42; SD = 0.70), sharing PMTCT evidence from research with multidisciplinary team (*M* = 0.47; SD = 0.77), and reading and critically appraising PMTCT clinical research (*M* = 0.49; SD = 0.79).

### Source of evidence for prevention of mother-to-child transmission practice

The respondents were further asked what source of evidence they use for PMTCT practice. The results indicate that the majority of nurses (70.4%; *n* = 57) mainly rely on college training, followed by in-service training 65.4% (*n* = 53), local policies and guidelines 60.5% (*n* = 49), personal experience 59.3% (*n* = 48), and policy initiatives and guidelines 58% (*n* = 47). Articles published in research journals, nursing journals and medical journals, the Internet and local audit reports were the least often consulted sources (Table 2).

**TABLE 2:** Sources of knowledge for prevention of mother-to-child transmission practice.

Sources of knowledge	Seldom		Frequently	
	
	*n*	%	*N*	%
Information that I learn about each patient/client as an individual	39	48.8	41	51.3
My intuitions about what seems to be right for the patient/client	46	57.5	34	42.5
My personal experience of caring for patients/clients over time	33	40.7	48	59.3
What has worked for me for years	53	66.3	27	33.8
The ways that I have always done it	52	65.0	28	35.0
Information my fellow practitioners share	41	50.6	40	49.4
Information senior clinical nurses share, for example clinical nurse specialists and nurse practitioners	41	51.3	39	48.8
What doctors discuss with me	44	55.0	36	45.0
New treatments and medications that I learn about when doctors prescribe them for patients	39	48.1	42	51.9
Medications and treatments I gain from pharmaceutical or equipment company representatives	48	59.3	33	40.7
Information I get from product literature	55	67.9	26	32.1
Information I learn in my training	24	29.6	57	70.4
Information I get from attending in-service training/conferences	28	34.6	53	65.4
Information I get from local policy and protocols	32	39.5	49	60.5
Information I get from national policy initiatives/guidelines	34	42.0	47	58.0
Information I get from local audit reports	65	80.2	16	19.8
Articles published in medical journals	70	86.4	11	13.6
Articles published in nursing journals	71	87.7	10	12.3
Articles published in research journals	71	87.7	10	12.3
Information in textbooks	50	61.7	31	38.3
Information I get from the Internet	66	81.5	15	18.5
Information I get from the media (magazines, TV)	62	77.5	18	22.5

### Interrelationships between knowledge, attitudes, practices and sociodemographics

Spearman’s rho correlation was performed to test the associations between the respondents’ knowledge, attitudes and practices. The results showed no linear relationship between attitudes and knowledge with a correlation coefficient value of 0.146, attitudes and practices with a correlation coefficient value of 0.083, and knowledge and practices with a correlation coefficient value of 0.320.

The Kruskal-Wallis and Mann-Whitney U-tests were performed to test for association between sociodemographic variables and knowledge, attitudes and practices. Knowledge was found to be positively associated with nursing category and qualifications, with *p*-values of 0.020 and 0.015, respectively. Attitudes were found to be associated with EBP training with a *p*-value of 0.045. Practice was found to be positively associated with age, *p* = 0.003, and experience, *p* = 0.043.

### Barriers to and facilitators of evidence-based practice in prevention of mother-to-child transmission

The main barriers that were identified by the nurses were insufficient resources (67.9%; *n* = 55), difficulties in accessing research reports (55.6%; *n* = 45), not knowing how to find appropriate research reports (51.9%; *n* = 42), lack of time (45.7%; *n* = 37) and difficulties in identifying implications of research findings to clinical practice (40.7%; *n* = 33).

The majority of the respondents, 88.9 % (*n* = 72), agreed that mentoring would facilitate EBP in PMTCT. Similarly, 84% (*n* = 68) agreed that adequate training was a facilitator of enhancing EBP application in clinical situations. Other facilitators included access to comprehensive literature, availability of time, support from colleagues and management (Table 3).

**TABLE 3:** Barriers to and facilitators of evidence-based practice in prevention of mother-to-child transmission.

Item	Agree		Neutral		Disagree	
		
	*n*	%	*n*	%	*n*	%
**Barriers**						
Insufficient resources to change practice	55	67.9	9	11.1	17	21.0
Research reports not easy to find	45	55.6	9	11.1	27	33.3
Do not know how to find research reports	42	51.9	17	21.0	22	27.7
Insufficient time to find research reports	37	45.7	15	18.5	29	35.8
Find it difficult to identify implications of research	33	40.7	18	22.2	30	37.0
Insufficient time to find organisation information	32	39.5	10	12.3	39	48.1
Organisation information not easy to find	32	39.5	4	4.9	45	55.6
Find it difficult to identify implication of organisation Information	30	37.0	22	27.2	29	35.8
Lack of authority in the workplace to change practice	29	36.3	11	13.8	40	50.0
Do not know how to find organisation information	28	34.6	11	13.6	42	51.9
Do not feel confident in judging quality of research	26	32.1	21	25.9	34	42.0
Culture of team not receptive to change	23	28.4	12	14.8	46	56.8
Find it difficult to understand research reports	21	25.9	19	23.5	41	50.6
Do not feel confident about changing practice	14	17.3	7	8.6	60	74.1
**Facilitators**						
Mentoring by nurses with adequate EBP experience	72	88.9	4	4.9	5	6.2
Given adequate training in EBP	68	84.0	8	9.9	5	6.2
Access to system for comprehensive literature review	66	81.5	10	12.3	5	6.2
Given protected time to conduct EBP	62	77.5	11	13.3	7	8.8
Nursing colleagues who embrace EBP	57	70.4	13	16.0	11	13.6
Nursing management who embrace EBP	53	65.4	18	22.2	10	12.3

EBP, evidence-based practice.

### Validity and reliability

Content validity was used for the study. This was done by relating objectives of the study to the specific questions on the instrument. A test-retest method was applied to assess the reliability of the self-completion questionnaire. The test-retest results were analysed using the Kappa statistic, which found an acceptable reliability coefficient ranging from 0.8 to 0.9. Cronbach’s alphas for the different sections of the questionnaire were as follows: knowledge or skills, 0.89; attitude, 0.69; practice, 0.92; barriers, 0.71; facilitators, 0.82. Acceptable Cronbach’s alphas were also found by the original authors of the instrument.

## Discussion

It is noteworthy that the response rate for the present study was very high at 94%. The results showed that the majority of the respondents were younger than 40 years, which is similar to previous studies (Dalheim et al. 2012). Just as nursing is said to be a female-dominated profession (Thompson, Chau & Lopez 2006), the majority of the participants in the present study were female. Linton and Prasun (2013) assert that nurses need to be trained at higher levels where EBP concepts are included in the curriculum. However, in the present study, the majority of the respondents (70.4%; *n* = 57) were trained below first-degree level.

The findings regarding PMTCT training for the nurses indicate that the majority of the nurses (70.4%; *n* = 57) were trained in PMTCT. This contrasts with the findings of a Nigerian study, where deficits were noted in the nurses’ knowledge and PMTCT practices because of lack of training as only 43% of their respondents had been trained (Ogbolu et al. 2013). Most of the current study’s respondents (73.7%: *n* = 42) who had been trained had received in-service training, which is consistent with previous studies’ findings (Zuber et al. 2014). The PMTCT training, however, is usually a once-off activity (Chopra et al. 2009; Zuber et al. 2014) and with the evolving nature of the PMTCT field, EBP is essential in preventing MTCT of HIV (Govender & Coovadia 2014).

Brown et al. (2009) argue that the majority of nurses are not adequately prepared for EBP. Similarly, a considerably large number of respondents (50.6%; *n* = 41) in the present study were not trained about EBP principles and, consequently, the majority (67.9%; *n* = 53) possessed average knowledge of EBP. The results of the study further portrayed that the majority of respondents (64.2%: *n* = 52) had poor research skills. These results corroborate previous findings in Australia, where lack of knowledge of basic aspects of research and how to translate research into practice were cited as reasons for low research use among nurses (Breimaier, Halfens & Lohrmann 2011).

Several previous studies have demonstrated that nurses generally have favourable attitudes towards EBP (Majid et al. 2011; Stokke et al. 2014). Similarly, the results of the present study showed that most of the respondents (64.2%; *n* = 52) had positive attitudes towards EBP in PMTCT. The majority of the nurses (59.3%; *n* = 48), however, were in agreement with the fact that their heavy workload prevented them from keeping up with new evidence. Previous studies also identified heavy workloads and lack of time as major barriers to implementing EBP (Brown et al. 2009; Majid et al. 2011).

The results of the current study showed that the nurses implemented EBP to a limited extent which is in line with previous studies’ reported findings (Brown et al. 2009; Stokke et al. 2014). Brown et al. (2009) found knowledge of EBP to be correlated with practice. The findings of the current study appear to contradict this finding as no association was found between knowledge and practice with a correlation coefficient value of 0.320. Other studies, unlike this study, also found practice to be associated with attitudes (Majid et al. 2011).

The ranking of barriers to EBP in the current study differs from most of the previous studies that ranked lack of time and lack of authority to implement new ideas as the major barriers (Brown et al. 2009; Majid et al. 2011) while respondents in the present study perceived lack of resources and difficulties in accessing research results as major barriers. There were many neutral responses on the barriers scale. Majid et al. (2011) report similar results because the nurses were not practising EBP; hence they were not aware of the barriers. The same conclusion can be drawn for the current study as the practice scale received the lowest score. The nurses considered mentoring, training and availability of time as major facilitators of EBP in PMTCT. This corroborates previous studies’ findings (Majid et al. 2011). Stokke et al. (2014) contend that opportunities should be made available for nurses to access journals when EBP is being introduced. Similarly, the current study’s respondents agreed that access to a system for comprehensive literature searching would facilitate EBP in PMTCT.

## Limitations of the study

The data were collected through the use of self-completion questionnaires, which might have been subject to personal bias and participants’ ability to assess their skills and practices.

The study was conducted at only one institution, and therefore the results need to be interpreted with caution and not be generalised to the whole population of PMTCT nurses in Malawi.

### Recommendations

Based on the findings of the study which demonstrated the respondents had (1) lack of access to resources to facilitate EBP utilisation such as research articles and access to research databases, (2) knowledge deficit in terms of research and its implication to clinical practice and (3) lack of time to find and utilise research reports, the following recommendations are suggested to improve EBP within an evolving area of nursing, namely PMTCT:

- The use of learning platforms such as peer-supported groups through establishing weekly journal clubs as a means to facilitate the use of new evidence and research reports specific to the area of PMTCT practice.
- Creating opportunities that support ongoing training and development which can be facilitated by nurse managers through in-service training that specifically addresses nurses’ knowledge gaps on how to use EBP such as the introduction and discussion of new PMTCT policies and research evidence on PMTCT practices.
- The use of peer mentoring is another strategy that can enhance EBP utilisation among PMTCT nurses. The use of learning spaces in the form of journal clubs or weekly discussion groups will enable PMTCT nurses to become sensitised to new available evidence related to their field of nursing.

## Conclusion

The findings of this study support and add to previous studies’ findings that nurses have positive attitudes towards EBP, but they are not adequately trained for successful implementation of EBP. This study, unlike previous studies, found no relationship between knowledge, attitudes and practices although some demographic factors were identified that influenced the nurses’ knowledge, attitude and practices. The study also found that EBP was hampered to a large extent by organisational barriers that the nurses face. Thus, EBP is a multidimensional construct and, to promote it, an effort should be made to address all influencing factors.
